# Fatigue modulates dopamine availability and promotes flexible choice reversals during decision making

**DOI:** 10.1038/s41598-017-00561-6

**Published:** 2017-04-03

**Authors:** Pierpaolo Iodice, Claudio Ferrante, Luigi Brunetti, Simona Cabib, Feliciano Protasi, Mark E. Walton, Giovanni Pezzulo

**Affiliations:** 1 Institute of Cognitive Sciences and Technologies, National Research Council, Via San Martino della Battaglia 44, 00185 Rome, Italy; 2 Department of Pharmacy, University G. d’Annunzio of Chieti, via dei Vestini 31, 66013 Chieti, Italy; 3 University Sapienza, Dept. Psychology, Centro D. Bovet, 00185 Rome, Italy; 4 CeSI-Met - Center for Research on Ageing and Translational Medicine & DNICS - Department of Neuroscience, Imaging and Clinical Sciences, University G. d’ Annunzio of Chieti, Chieti, 66100 Italy; 5 Department of Experimental Psychology, University of Oxford, Oxford, UK

## Abstract

During decisions, animals balance goal achievement and effort management. Despite physical exercise and fatigue significantly affecting the levels of effort that an animal exerts to obtain a reward, their role in effort-based choice and the underlying neurochemistry are incompletely known. In particular, it is unclear whether fatigue influences decision (cost-benefit) strategies flexibly or only post-decision action execution and learning. To answer this question, we trained mice on a T-maze task in which they chose between a high-cost, high-reward arm (HR), which included a barrier, and a low-cost, low-reward arm (LR), with no barrier. The animals were parametrically fatigued immediately before the behavioural tasks by running on a treadmill. We report a sharp choice reversal, from the HR to LR arm, at 80% of their peak workload (PW), which was temporary and specific, as the mice returned to choose the HC when the animals were successively tested at 60% PW or in a two-barrier task. These rapid reversals are signatures of flexible choice. We also observed increased subcortical dopamine levels in fatigued mice: a marker of individual bias to use model-based control in humans. Our results indicate that fatigue levels can be incorporated in flexible cost-benefits computations that improve foraging efficiency.

## Introduction

In both the wild and the laboratory, animals’ preferences for one course of action over another reflect both the value of action goals and the cost or effort that must be invested in pursuing them. *Effort-based decision making* provides a test situation to investigate how we make an action choice based on an integration of goal values and action costs^1, 2^. For example, several studies have shown that animals will choose to put in effort (e.g. make choices that require climbing a barrier to secure a reward), and avoid a less effortful option, if the gains are sufficiently large^2, 3^. Lesions to the anterior cingulate cortex or of the dopamine projection to the nucleus accumbens can cause cost-aversion and therefore preference reversals in these paradigms^3–5^. However, relatively little is known concerning the way metabolic fatigue (e.g. due to physical effort or a demanding physical exercise) influences effort-based choices, and whether animals can flexibly incorporate information on the physiological state of their body (here, fatigue) within cost-benefit analysis. Here, we asked if changes in physiological state, i.e., fatigue, determine a fast re-evaluation of cost-benefits prior to the choice - which would imply an immediate re-adaptation to the novel task contingencies - or a slow re-adaptation process that implies trial-and-error learning^6–10^.

Consider the case of a rodent in a T-maze choosing between a high-reward option (HR) that can only be obtained at high cost by climbing over a barrier, and a low-reward, low-cost option (LR) where the reward is available in an otherwise unoccupied arm of the maze. Existing approaches to (mental and physical) fatigue suggest contrasting predictions in this task. One stream of research reveals that, when a cognitive task and a physical exercise are performed together, the cognitive performance is generally impaired (“dual task” effect) in a way that increases with the energetic constraints of the task^11^. In keeping, one should expect that physical effort might generically impair choice performance (e.g., make it more random). Another stream of research reveals that mental effort induces humans to use less demanding, automatic or habitual (model-free) control strategies rather than more demanding and more flexible (model-based) strategies based on cognitive planning^12^. According to this hypothesis, animals trained in an effort-based task without fatigue should keep selecting their most-preferred option (HR) even when they are later fatigued, at least for a few trials. Choice reversal from HR to LR would thus require a slow (trial-and-error) process.

An alternative perspective is that animals may use their fatigue level as a source of information, along with reward expectation, to perform a *cost-benefit analysis* prior to the choice^13–15^. Because fatigue information enters in the decision phase, one should expect an immediate change in the choice pattern, from HR to LR, when the level of fatigue is sufficiently high. In other words, the level of fatigue (playing the role of a “decision boundary”) enters in the cost-benefit analysis and, at a sufficient level, will produce a sharp and rapid preference reversal. Furthermore, the cost-benefit analysis hypothesis would predict an opposite preference reversal (from LR to HR) when fatigued animals face a double-barrier task, which imposes the same costs on both branches. Of note, this normative (cost-benefit) perspective has to be carefully teased apart carefully from a seemingly related possibility: that fatigue affects decision-making by inducing Pavlovian avoidance/aversion bias^16, 17^ (e.g., a bias to avoid costly actions such as climbing barriers). Like the former (cost-benefit) view, this alternative (Pavlovian) view predicts that fatigued mice should reverse their choice pattern immediately; but it also predicts that they should avoid climbing barriers independent of the arm where they are placed - or in other words, that animals should not be fully sensitive to the balance of costs and benefits.

To test the predictions of these contrasting views, we parametrically varied the level of fatigue (from “no fatigue” to 40%, 60% and 80% of maximal workload of each specific animal) of a population of mice that performed a series of effort-based T-maze tasks (HR vs. LR) with one barrier on the HR branch, and a double-barrier task with barriers on both HR and LR branches^18^.

In addition, we asked whether experiences leading to different choices induce changes in brain dopamine (DA) and serotonin (5-HT) - two amines that are differently involved in effortful choice^19^. To this aim, we evaluated DA and 5-HT tissue levels in the medial prefrontal cortex, nucleus accumbens, midbrain and hippocampus of fatigued mice that performed the decision task, fatigued mice that did not perform the decision task, and non-fatigued mice that performed the decision task.

## Results

### Experiment 1: Effort-based decision making under various levels of fatigue

**Figure 1:**
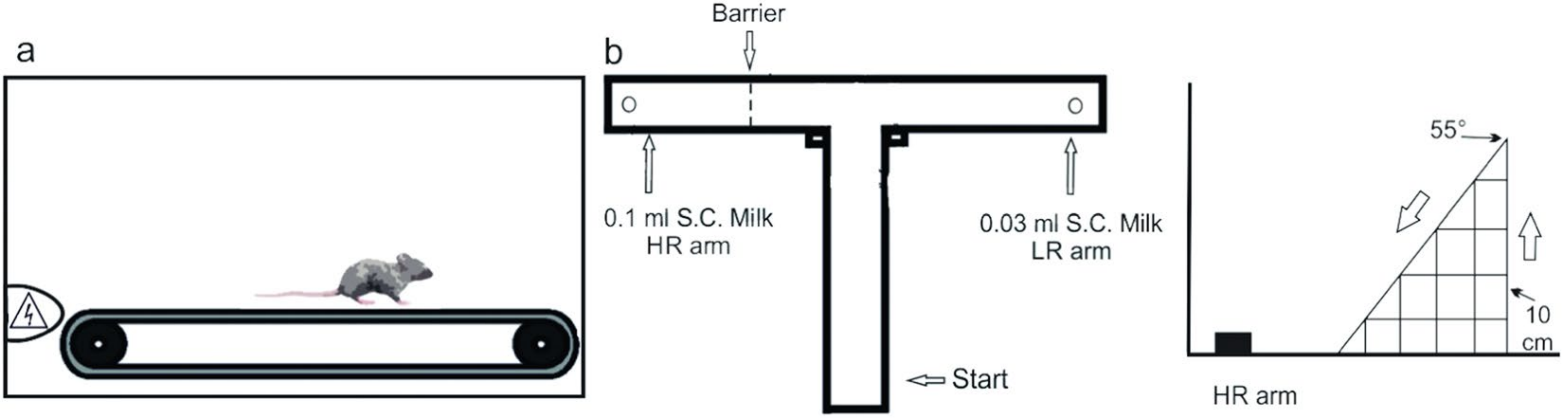
Experimental apparatus. (**a**) Treadmill schematic side view used throughout the running period of the study. The humidity and temperature of treadmill chamber were constant during each session. (**b**) T-maze schematic top view, mice were placed in the start arms and allowed to choose between the two arm goals. To obtain 0.1 ml of sweetened condensed milk, the animals could climb over a wire mesh barrier (HR arm); otherwise, mice could choose the low reward arm (reward = 0.03 ml). In experiment 4, a second barrier was also placed in the LR arm.

Eight female C57BL/6 mice (Exp group) were tested in an effort-based decision-making task consisting in choosing one of two arms of a T-maze^4^: an arm baited with a small reward (LR: low reward/no effort) or an arm baited with a higher reward but which required climbing a 10 cm barrier (HR: high reward/high effort). The mice did 8 trials every day. They were tested under 4 levels of fatigue: no fatigue (block A, 3 days), 40% of their peak workload (PW) (block B, 3 days), 60% PW (block C, 3 days), and 80% PW (block D, 3 days), see Fig. 1. Fatigue was induced with 40 min of continuous run on a motor treadmill. Peak workload was specific for each animal.

**Figure 2:**
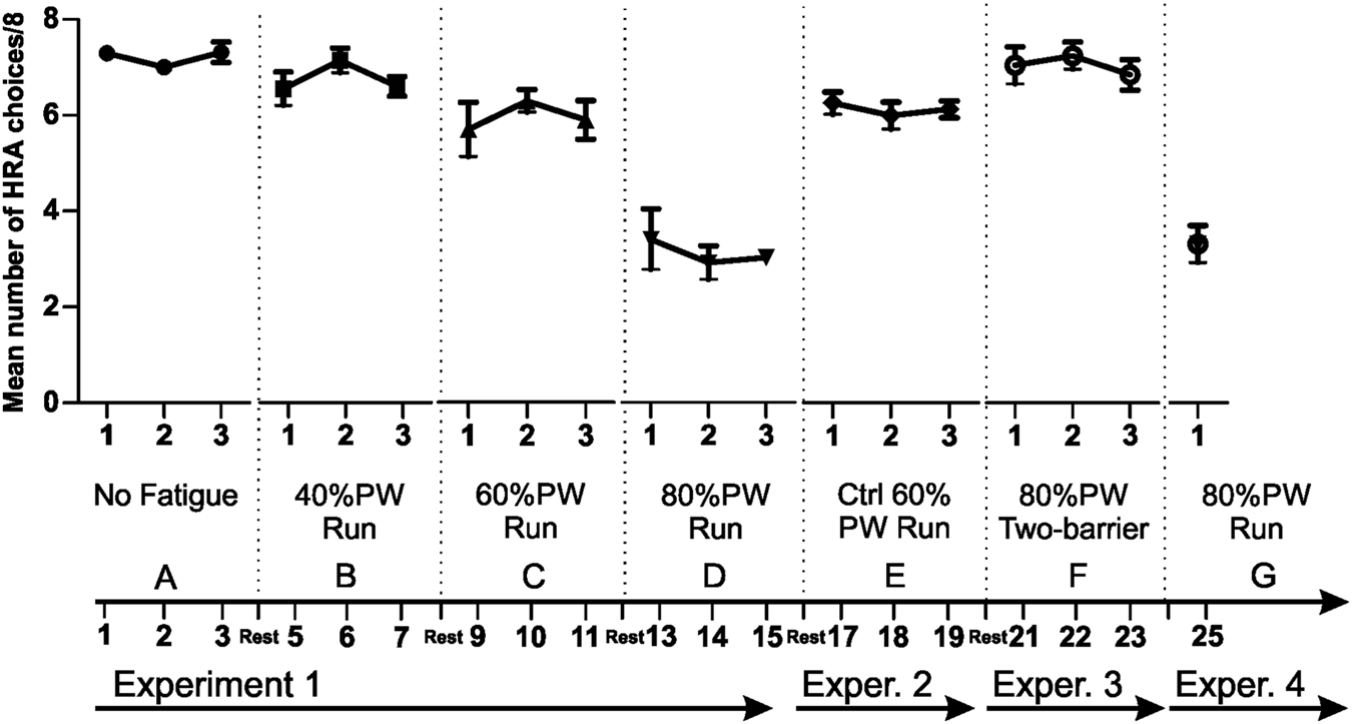
Results of the Effort-Based Decision Making task. The figure reports the behavioural performance of the Experimental group across the three decision experiments, as the mean (±SEM) number of trials in which mice chose the high effort HR arm. Experiment 1 was composed of four blocks (A–D), each consisting of eight trials. A mesh 10-cm barrier was placed in the HR arm. Immediately before the behavioural tasks the animals performed a 40 min run on a mechanical treadmill. The intensity of physical effort was parameterized on maximal power load (PW) of each animal. Intensity of run was improved each block with this progression: negligible (block A); 40%PW (block B); 60%PW (block C); 80%PW (block D). (Note that mice in the CtrlDec group also performed block A, with the same performance as the Experimental group). In experiment 2, the intensity of the run period was decreased to 60%PW as in block C. In experiment 3, there were 10-cm barriers in both arms. In experiment 4, the procedure was the same as block D, but for only 1 day.

The mean number of high reward/high effort arm choices in the different blocks is displayed in Fig. 2. To analyse the results the data were subjected to repeated-measures ANOVAs with two within-subjects factors: testing block (4 levels: “block A = No fatigue”, “block B = 40% PW”, “block C = 60% PW” and “block D = 80% PW), and day of testing (3 levels: “day 1”, “day 2”, and “day 3”). The dependent variable was the sum of HR arm choices.

**Figure 3:**
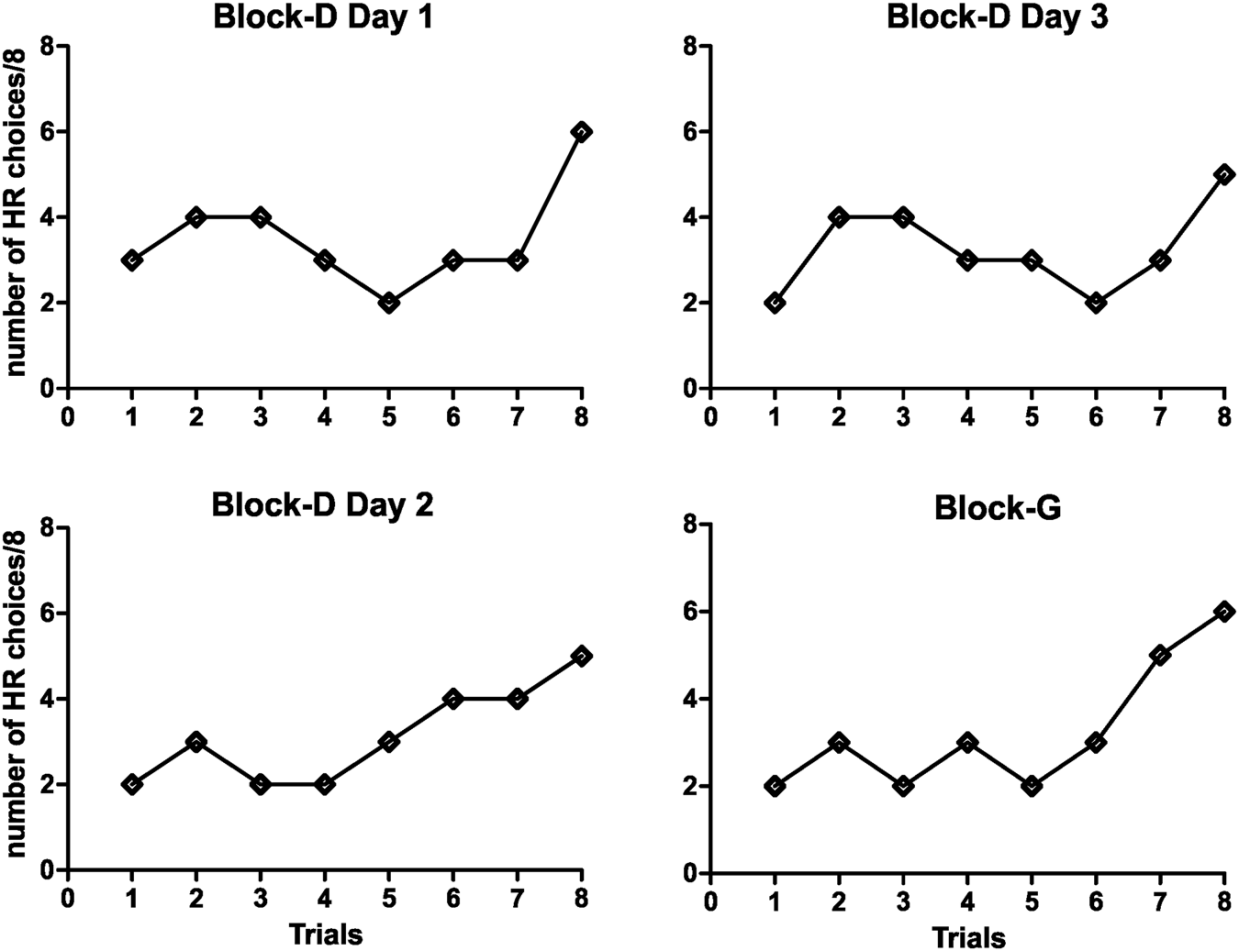
Behavioural strategy changes were evident from the first trial. When highly fatigued (blocks D and F), mice of the Exp group showed a clear preference for LR (thus, a reversal of choice from HR to LR) from the very first trial every day, corroborating the idea that they were performing a cost-benefit computation rather than learning to avoid the costly HR choice by trial and error. The graph also shows that this preference for LR was maintained (on average) until the 8^th^ trial, suggesting that this was a good window to investigate the phenomenon.

There was a significant effect of block (*F*_3,42_ = 87.844, p < 0.0001), but not of day (*F*_2,22_ = 0.591, NS). There were no significant effect of block × day interaction, (*F*_6,84_ = 0.601, NS). Bonferroni’s post hoc test showed that these effects can be explained by the difference in performance on the Block D (fatigue 80% PW) and all other block conditions. We found no significant differences between blocks A vs. B (NS), A vs. C (NS), or B vs. C (NS). When the fatigue reached the 80% of maximal power load, animals chose the high effort HR arm significantly less often than in other blocks (p < 0.01) (Fig. 2). Looking at the trial-by-trial choices of Exp group mice in Block D, the behavioural change of preference from HR to LR is evident from the very first trial of every day (see Fig. 3), further corroborating the idea that it is part of a (faster) cost-benefit strategy, not the effect of (slower) trial-and-error learning.

### Experiment 2: Re-test in the medium fatigue condition (60% of PW)

To determine how sensitive the mice’s decision policies were to the currently experienced level of fatigue, the 8 mice of the Exp group were tested again at 60% fatigue (block E, 3 days) - thus, with a level of fatigue that was again lower than the last block of Experiment 1 (block D, 80% PW), and the same as the penultimate block of Experiment 1 (block C, 60% PW). A repeated-measures ANOVA (blocks × days of testing) of HR arm choices sum showed a significant main effect of blocks (blocks C, D, E - *F*_2,63_ = 60.490, p < 0.0001), no main effect of days of testing (*F*_2,63_ = 0.057, NS), and no significant interaction (*F*_4,63_ = 0.613, NS). Animals changed in the frequency of HR arm preferences (Post-hoc analysis, block D vs. E, t = 5.368, p < 0.001), returning to choose to climb the barrier to gain the high reward on the majority of trials. Importantly, this reproduced the same behaviour manifested in block C (Post-hoc analysis, block E vs. C, t = 0.507, NS), showing the kind of behavioural flexibility that is a hallmark of cost-benefit calculations.

### Experiment 3: Double-barrier task in high fatigue condition (80% of PW)

The results of previous experiments might be dependent on the fact that fatigue makes the mice physical incapable of climbing the barrier or renders the mice insensitive to reward magnitude differences, rather than changing the cost-benefit calculations. To rule out these possibilities, in Experiment 3, the 8 mice of the Exp group were tested with a second barrier placed in the low reward arm, so that the level of effort was the same for both arms (block F) when they had a high (80% PW) level of fatigue. This caused the mice to choose the HR arm on almost all trials. Two-way ANOVA analysis of two blocks (D and F) for the 3 days of testing revealed a main effect of task (single barrier versus double barrier control, *F*_1,42_ = 164.57, p < 0.0001). These results indicate that when highly fatigued animals had to invest a high level of physical effort (i.e., the barrier was present on both arms) to secure *either* reward, the mice preferred the HR.

### Experiment 4. Re-test in the high fatigue condition (80% of PW)

Finally, the 8 mice of the Exp group were tested again at 80% of PW (block G) for a single day. We found no significant differences from block D (NS). The aim of this experiment was twofold: first, to provide converging evidence that a high fatigue condition (80% of PW) can induce preference reversals from HR to LR; second, and most important, to successively assess the effect of high fatigue on dopamine (DA) and serotonin (5-HT) levels.

For this, we compared DA and 5-HT levels in the nucleus accumbens, hippocampus, midbrain and frontal cortex, of the Exp group (n = 8), which performed Experiments 1–4, and two additional control groups: CtrlDec (n = 8) and CtrlRun (n = 4). The Exp group animals were sacrificed immediately after their T-maze choice session of Experiment 4 (i.e., after high fatigue: 80% of PW - a condition that we have shown to induce a choice reversal from HR to LR choices). The CtrlDec animals followed the procedure of Experiment 1, block A (a low intensity running on a treadmill, not inducing fatigue, and T-maze testing) and where sacrificed immediately after. Finally, the CtrlRun animals followed the same run procedure of Exp group animals throughout Experiments 1–4, but not the T-maze choice sessions. They were sacrificed immediately after the procedure described in Experiment 4 (but without the T-maze choice session).

**Figure 4:**
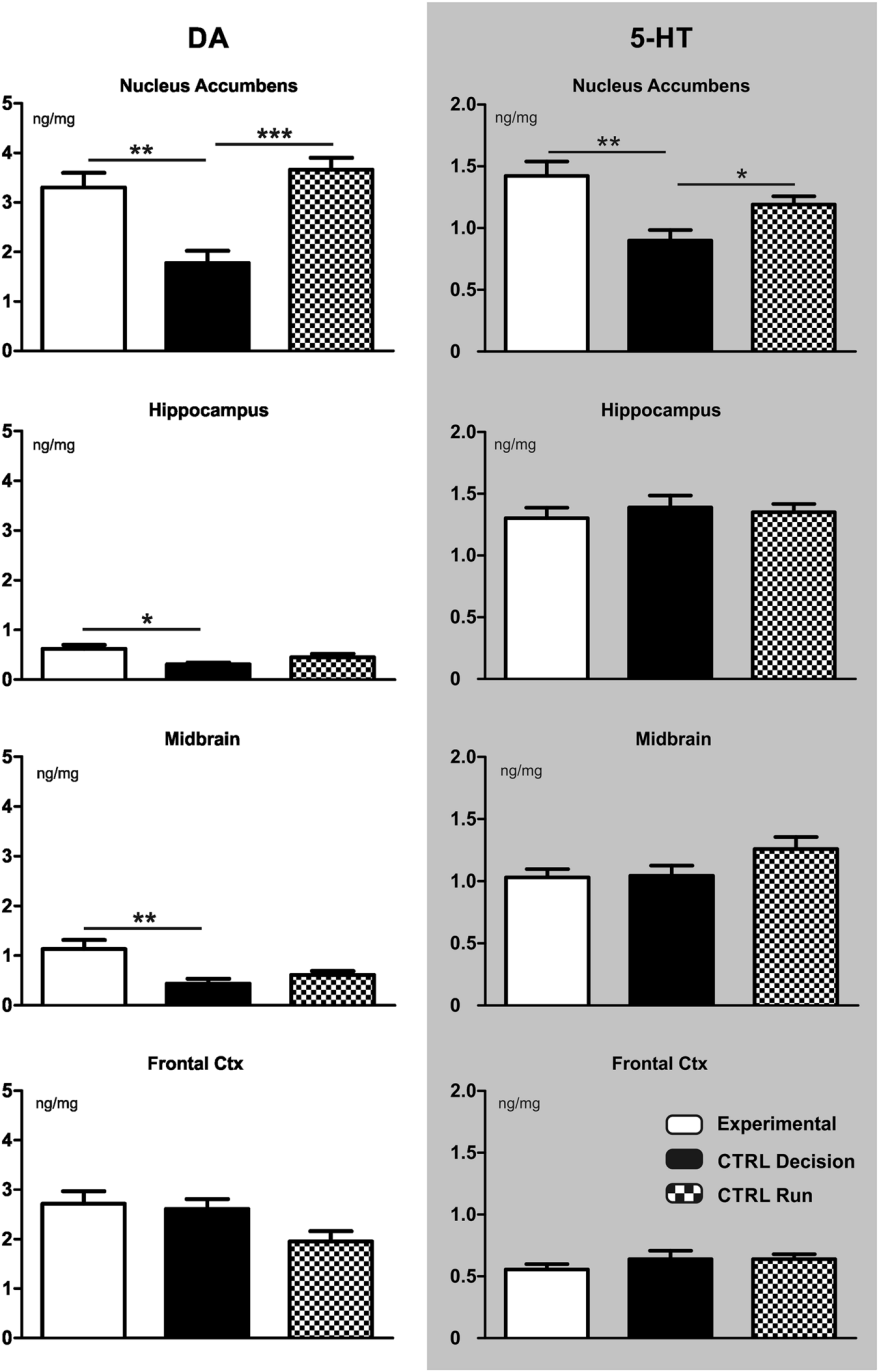
Effects of fatigue on dopamine and serotonin levels. Tissue levels of serotonin (5-HT) and dopamine (DA) (ng/mg tissue; mean ± SEM) of three mice groups: Exp (Experimental: performed 40 min run on treadmill at 80% of their maximal power load, and then the T-maze choice), CtrlDec (Control Decision: performed 40 min run on treadmill at low intensity, and then the T-maze choice), and CtrlRun (Control Run: performed 40 min run on treadmill at 80% of their maximal power load, but not the T-maze choice). Tissue levels were analysed in four brain areas: Frontal cortex, hippocampus, midbrain and nucleus accumbens. Post-hoc analysis significance: *p < 0.05; **p < 0.01; ***p < 0.001. Note that DA levels in the frontal cortex - which are generally in an inverse relationship with DA levels in the accumbens^43^ - were higher in all three groups compared to naive mice (0.15 ng/mg in a group of 4 naive female C57BL/6 mice we tested) but this activation did not significantly vary across the conditions we studied.

Tissue concentrations of 5-HT and DA (ng/mg tissue; mean ± SEM) in the different areas sampled were reported in Fig. 4.

To determine whether any significant differences existed in DA and 5-HT levels, we conducted four one-way ANOVAs between three different conditions (Exp, CtrlDec and CtrlRun groups), one in each observed brain area: nucleus accumbens (NAc), hippocampus (Hipp), midbrain (MB) and frontal cortex (FC). The main effect were found to be significant in all the brain areas sampled but in the prefrontal cortex, which was however close to significance (NAc: *F*_2,21_ = 14.74, p < 0.001; Hipp: *F*_2,21_ = 5.949, p < 0.01; MB: *F*_2,21_ = 8.090, p < 0.01; FC: *F*_2,21_ = 3.435, p = 0.0512). Post-hoc analysis revealed a significant effect of the Exp versus CtrlDec condition on DA levels (NAc: q = 6.034, p < 0.01; Hipp: q = 4.664, p < 0.01; MB: q = 5.820, p < 0.01) and a significant change of DA level in the CtrlRun group in NAc area (q = 7.228, p < 0.001). In other words, NAc DA levels were higher after fatigue, whereas in MB and Hipp DA increased if mice were both fatigued and performed the decision task. Overall, these results suggest that the experimental condition we studied induces an adaptation of the mesolimbic DA system, and imply running fatigue on DA augmentation. Furthermore, DA increase in MB and Hipp appears to be an interaction of fatigue and decision.

As for 5-HT, statistical analyses only revealed a significant main effect of the experimental manipulation in the NAc (*F*_2,21_ = 8.118, p < 0.01), further supporting the role of this brain area in adapting to fatigue.

A one-way ANOVA analysis of DOPAC/DA ratio for each observed brain area was performed, in order to determine any significant differences between the three experimental conditions (Exp, CtrlDec and CtrlRun). The main effects were found to be significant in all the brain areas (NAc: *F*_2,21_ = 19.552, p < 0.01; Hipp: *F*_2,21_ = 21.513, p < 0.01; MB: *F*_2,21_ = 510.631, p < 0.0001; FC: *F*_2,21_ = 6.208, p < 0.05). See Table 1 for post-hoc significance (using Bonferroni correction).

**Table 1:** DOPAC/DA ratio.

DOPAC/DA Ratio	Experimental	Ctrl Decision	Ctrl Run
NAc	0.13 ± 0.06^#^	0.43 ± 0.18^†^	0.09 ± 0.08
Hippocampus	0.61 ± 0.11^†^	1.01 ± 0.22^†^	0.38 ± 0.23
Midbrain	0.59 ± 0.05^†,#^	2.29 ± 0.12^†^	0.23 ± 0.20
FC	0.16 ± 0.08^†^	0.13 ± 0.06	0.05 ± 0.05

Bonferroni’s post-hoc analysis significance: ^#^p < 0.01 vs. CtrlDec; ^†^p < 0.01 vs. CtrlRun.

A one-way ANOVA analysis of 5-HIAA/5-HT ratio for each observed brain area was performed, in order to determine any significant differences between the three experimental conditions (Exp, CtrlDec and CtrlRun). Our data showed significant main effects in all brain areas (NAc: *F*_2,21_ = 92.462, p < 0.001; Hipp: *F*_2,21_ = 37.224, p < 0.001; MB: *F*_2,21_ = 145.733, p < 0.0001; FC: *F*_2,21_ = 82.751, p < 0.001). See Table 2 for post-hoc significance (using Bonferroni correction).

**Table 2:** 5-HIAA/5-HT ratio.

5HIIA/5HT Ratio	Experimental	Ctrl Decision	Ctrl Run
NAc	1.21 ± 0.09^#,†^	0.62 ± 0.11	0.58 ± 0.11
Hippocampus	0.87 ± 0.11^#,†^	0.55 ± 0.18^†^	0.31 ± 0.08
Midbrain	1.32 ± 0.18^†^	1.34 ± 0.13^†^	0.21 ± 0.14
FC	1.24 ± 0.21^#,†^	0.65 ± 0.11^†^	0.26 ± 0.12

Bonferroni’s post-hoc analysis significance: ^#^p < 0.01 vs. CtrlDec; ^†^p < 0.01 vs. CtrlRun.

## Discussion

Many classical and contemporary studies have assessed the effect of effort expenditure on response rates of humans and other animals (e.g., rodents), by varying experimental parameters such as the weight of a lever to be pressed, the height of a barrier to be climbed, or the number of handle turns needed to generate a unit of reward^2, 20–26^. These and other studies have shown that humans and other animals are typically able to evaluate effort effectively and rapidly - where effort may be estimated via the metabolic energy consumed to produce an action^27^ - and tend to maximize gain whilst minimizing energy expenditure^28, 29^. For example, starlings choose between walking or flying depending on optimal solution of net gain between energy requirements in walking or flying and the food gained^28^. Laboratory studies show that, if reward magnitude is held constant, then high-effort tasks tend to be avoided^22^ and convergent evidence has been reported in rodent and non-human primate studies as well^2, 3, 18, 30, 31^. Despite this progress, relatively little is known on how the physiological condition of the body - and in particular physical fatigue - affects effort-based choice.

We report that, by manipulating the levels of fatigue of mice, we were able to induce sharp and rapid preference reversals from HR to LR and vice-versa in effort-based choices. Our results indicate that a high level of fatigue (Experiment 1, block D and Experiment 4) induces a sharp preference reversal, from HR to LR choices. Returning to a less severe fatigue level (Experiment 2) induces the opposite preference reversal, from LR to HR. This result is even stronger when animals face a double-barrier task (Experiment 3). These preference reversals are reliable and fast (i.e., occur from the very first trial) – an index of behavioural flexibility.

When animals are very fatigued (here, 80% of their individual PW) and face a barrier, the effort required to climb the barrier surpasses the benefits of the HR. Importantly, this situation does not correspond to the physical impossibility of climbing the barrier, as the mice in fact climbed the barrier in the two-barrier condition, when it was required by the task. Furthermore, an analysis of mice behaviour reveals that they clearly showed their preference for the HR or the LR at the decision point, rather than (say) firstly going in the direction of the HR and successively revising their choice. This latter behaviour was impossible in our set-up, as mice were prevented from changing branches after the decision point.

These results point to the conclusion that mice considered the (fatigue-scaled) effort to be made in a flexible *cost-benefit* process prior to the choice, without the necessity to re-learn how to behave in each novel situation by trial-and-error. In particular, the fast preference reversals from HR to LR and vice versa when physiological conditions (i.e., fatigue) changed appear to be part of a strategy that balances action values and costs/effort flexibly and rapidly. Accordingly, our results indicate that information on motor costs and the physiological condition of the body (here, fatigue) can be incorporated into cost-benefit analysis, to optimize behaviour^13, 15, 32–34^ and run against the alternative views that it might generically impair choice (e.g., making it more random) or promote stereotypical responses (e.g., inducing a preference for the most-practiced or most-rewarded branch). A strong test for the “cost-benefit analysis” hypothesis is the fact that we were able to induce three successive preference reversals, and these were apparent from the first trial and survived for all the three days of testing. Furthermore, the results of the double-barrier task - i.e., the fact that animals made a choice requiring them to climb a barrier as reliably as in the former conditions, which required no barrier climbing - rules out the possibility that our results are due to the impossibility to climb barriers or a non-specific (aversive, Pavlovian) bias against high cost choices.

Additionally, we tested whether exposure to fatigue affected brain DA and 5-HT tissue levels and metabolism. While the ways DA regulates behaviour with regard to energy resources availability, nutrition and metabolic state have been widely studied^35–37^, here we focused instead on transient states of fatigue. The main finding of this experiment was the increase in tissue levels of DA in the NAc of mice exposed to the high fatigue protocol (both Experimental and CtrlRun, see Fig. 4), possibly due to a reduction of DA metabolism (reduced DOPAC/DA ratios, see Table 1). To interpret this finding properly, it is important to note that DA tissue level is a measure of overall, mostly presynaptic, DA availability^38^, not necessarily a measure of DA release, thus it does not permit to draw conclusions on the causal relationship between the catecholamine response and the observed behaviour. Nonetheless, results from human studies indicate a role for overall striatal DA availability in choice behaviour. Administration of L-DOPA, which increases DA synthesis, enhances flexible (*model-based*) over more automatic (*model-free*) choices in a sequential decision-making task^39^. Furthermore, unmedicated Parkinson patients characterized by low striatal DA availability show a selective impairment of *model-based* learning that is remediated by pharmacological replacement of DA availability in the same paradigm^40^, and DA modulation of NAc in medicated Parkinson patients has been shown to be specifically involved in choice behaviour rather than pure learning^41^. Finally, in healthy drug-free subjects high ventral striatal presynaptic DA levels are associated with bias toward *model-based* choice in a sequential decision-making task^42^. Together, these observations support the conclusion that increased availability of NAc DA could facilitate behavioural flexibility. Strenuous treadmill running may be stressful for animals, and stressors are known to engage the mesoaccumbens DA system^43, 44^. However, the metabolic pattern observed in the NAc of fatigued mice is different from that observed in stressed mice by previous studies^45–47^. Thus, mice exposed to up to 120 min of restraint; to 60 minutes of scrambled foot-shocks in either an escapabable or an inescapable paradigm; or to 10 minutes of forced swim show a significant increase of the DOPAC/DA ratio in the NAc with no alterations of DA tissue levels^45–47^. DA tissue levels in the PFC were higher than those reported in previous studies. None of the groups examined was made up of un-handled mice: the CtrlDec group, which was not exposed to the strenuous running, was extensively trained and tested in the food-reinforced T-maze. Moreover, all animals were under a restricted feeding protocol (see Materials and Methods). Therefore, high DA levels in the PFC could be the outcome of the specific experimental conditions used in this study.

We found significant increase of 5-HT tissue levels in the NAc, too. However, the 5-HIAA/5-HT ratio was increased in Exp group and not affected in CtrlRun. The changes in 5-HT levels paralleled DA - an interesting finding because 5-HT/DA interactions within the NAc play a main role in the control of impulsive choice^48^. To this regard, it is worth pointing out that 5-HT levels were highest in fatigued mice that performed the decision task and lowest in non-fatigued mice that performed the decision task, potentially in keeping with the recognized role of this neuromodulator in aversive situations^49^. Despite the limitations of our measures, it is tempting to speculate that modulations of serotonin may be part of an adaptive mechanism that regulates the cost-benefit trade-offs inherent in costly choices, so as to resist the pressing influence of HR. Some support for a role of serotonin in promoting deliberate over impulsive behaviour comes from evidence that high serotonin neuron firing facilitates rats’ waiting behaviour when rewards are delayed^50^ and that chronic 5-HT depletion makes animals more impulsive on a delay-based decision task^19^. Serotonin may have a more specific role in overcoming effort cost, too, at least in some circumstances^51^ (but see Yohn *et al*.^52^ for evidence that enhancement of 5-HT transmission by administration of fluoxetine reduces selection of high-effort lever pressing in rats tested on an effort-based choice task, and Denk *et al*.^19^ for evidence that serotonin blockers influence delay - but not effort-based decisions).

Our results can be interpreted within biologically-motivated models of choice in which *model-free* mechanisms of choice mediate decisions in well-known contexts, but more flexible *model-based* mechanisms are called upon when the choice context changes, such as for example when the usually rewarded branch in a T-maze is devaluated or task contingencies change^6, 9, 32, 53–58^. The rationale for invoking a model-based system after contextual changes is that it permits the choice offers to be evaluated anew, thus avoiding stereotyped behaviour that would be maladaptive. In our task, reward contingencies are stable, but fatigue changes action costs and the metabolic energy consumed to produce an action (e.g. climb a barrier) - hence the necessity to evaluate anew the choice offers. Furthermore, the fatigue procedure may increase the value of reward outcomes as rewards providing energy would cause a greater proportional improvement in energetic state^59^. A model-based system that permits the animal to predict action outcomes in a context-dependent way (here, fatigue-scaled rewards and costs) is ideal for this process of “re-evaluation-after-fatigue”. Note that for tasks that are not novel, the “model” may be quite minimalistic and only include context-specific associations (e.g., mappings between levels of fatigue and climbing costs) - what is essential is that it gives access to updated (context-specific) action values and costs to be used for cost-benefit computations, rather than promoting automatic responses that are not sensitive to changes in costs and benefits.

If the “re-evaluation-after-fatigue” hypothesis holds, one should expect increased DA levels in areas that are important for outcome valuation after the fatigue procedure. In keeping, our results show that the fatigue procedure alone is sufficient to increase DA levels in NAc - an area that is critical for goal-directed decision making^60^. In sum, an increased fatigue level - possibly signalled by interoceptive channels^61^ - might indicate to the animal that the choice context has changed, which in turn would favour a re-valuation of choice outcomes and the selection of a flexible decision strategy that balances costs and benefits and ultimately optimizes foraging efficiency. Increased DA and 5-HT levels may jointly promote behavioural flexibility by supporting model-based computations and preventing impulsive choices^19, 50^, respectively. It has been also proposed that increased DA and 5-HT levels during strenuous tasks may constitute a sort of reward for investment of effort^62^ or a way to mobilize the resources needed to face high-demanding tasks, even in the absence of immediate reward^63^ - and thus effortful choices (and strenuous exercise) would require high levels of DA and/or 5-HT, until a ceiling level is reached and the animal gives up. However, the relationships between strenuous tasks (or forced exercise) and DA/5-HT have not been systematically investigated so far and most animal studies are performed giving the animal free access to the wheels (a situation that is not comparable with our task). There is evidence of enhanced DA release during low-intensity treadmill exercise^64^, which was interpreted as an anti-nociception mechanism; but these findings are difficult to compare with the present results because, as discussed, the latter cannot be interpreted as indicating enhanced DA release. These and other hypotheses on DA and 5-HT modulations thus remain to be tested in future studies that measure (and manipulate) neuromodulator levels of animals under various levels of fatigue and effort.

In conclusion, our experiment provides evidence for the first time that information on the current physiological state of the body (fatigue) influences dopamine levels and can be incorporated in decision-making, promoting flexible forms of choice. Historically, the processing of information needed for decision-making and motor control were thought to operate sequentially, under the assumption that cognitive processing generates a decision and the outcome is then transformed into action by the motor system. Recently, the evidence regarding the feedback perturbations generated by the state of the motor system and by the muscular work rate during the process of decision have brought scientists to propose new models of effort-based decision-making. Nonetheless, few studies in the literature investigate the role of physical exercise and muscular fatigue in effort-based choice, and in particular whether fatigue influences decision processes or only post-decision action execution.

Here, by parametrically varying the levels of fatigue of mice in an effort-based decision-making task, we were able to produce sharp and reliable preference reversals, from high-reward to low-reward options, and vice versa, from the very first trial. Our results support the hypothesis that preference shifts depend on a flexible decision process involving cost-benefit computations, not a slow re-adaptation to the novel contingencies via trial-and-error learning. In other words, our results show that muscular fatigue can be incorporated in a flexible strategy for cost-effort considerations before a choice is made, rather than generically impairing choice or making it more stereotypical. Furthermore, we found changes in subcortical dopamine levels when fatigue reached a critical threshold. A high level of the neuromodulator dopamine in fatigued mice may represent a marker of individual bias to use flexible, model-based action choice, as reported in humans^39, 42^.

## Materials and Methods

### Animals

Twenty female C57BL/6 mice (Charles River Laboratories International, Inc., USA) were used for this study. All were housed in groups of four in a room maintained on a 12:12 light/dark cycle (lights on during the day) in a temperature-controlled environment (21 ± 1 °C). They were ~3 months old at the start of training period. All testing will occur during the light phase of the day. During experimental testing water was available ad libitum and they were food-restricted to reach 85% free feeding body weight throughout the study. All animals were under a protocol approved by the Animal Ethical Committee of University of Chieti and all experimental procedures complied with European Community Council directive (2010/63/EU). All surgeries were made to minimize animal suffering: animals were sacrificed by cervical dislocation as approved by the local University Committee on Animal Resources (15/2011/CEISA/COM).

### Apparatus

The T-maze protocol used for behavioural testing is based on that described by Salamone^4^ and Walton *et al*.^18^, but it has been modified for use by mice, see Fig. 1. The T-maze was a high-sided matt PVC T-maze, consisting of a start arm and two goal arms all of which were lined by walls, 20 cm high. The start arm joined on the two goal arms, all of which were 30 cm in length and 10 cm wide. A raised food well (1 cm in diameter) was fixed at the far end of each goal arm, equidistant from all sides. The walls of the maze were black while the floor was grey.

The barriers over which the mice will have to climb to obtain a reward in the chosen goal arm were made out of a heavy wire mesh bent to form a 3 dimensional right-angled triangle. The animals had to scale the vertical side but were able to descend down the slope of varying inclinations (in relation to the barrier size). Both a 5 cm and a 10 cm barrier were used during training. All tests were conducted with a 10 cm barrier.

### Incremental load test

The experimental procedures reported below include two kinds of tasks: a T-maze decision task (effort-based choice) and a run (fatigue) procedure. In the latter, the run velocity of each animal was specific to its own aerobic capacity. Thus, before all the experimental procedures, mice were submitted to incremental exercise testing on a motor treadmill.

The multiple-lane treadmill (Exer-3/6 treadmill, Columbus Instruments, Ohaio, USA) was placed in the climatic chamber for training. Stainless steel grids at the end of the lines provided an electrical stimulus of 0.25 mA, 1 Hz, and 200 ms length, to keep the mice running, and brushes prevented the mice from pinching feet between grid and treadmill. The intensity of exercise was increased by 3 meter/min (6–33 meter/min) every 3 min at 0% grade until exhaustion, which was defined as the point at which mice touched the end of treadmill five times in one minute. This test provided the total distance run and the peak workload (PW) for each animal, with the aim to determine aerobic capacity and exercise training intensity for each animal. Based on the latter, individual workloads corresponding to 40%, 60% and 80% peak workload (used in Experiments 1–3 below) were determined for each animal. Total distance run varies in a range between 385 to 411 m (mean = 394) and PW in a range between 18 to 21 m/min (mean = 20).

### Effort-based decision protocol

Habituation and training protocols were based on previous work^18^, which is briefly summarized below. The first week the mice were put on a restricted feeding schedule and were handled every day by the experimenter and habituated to the maze and treadmill. When they reached 85% of their free-feeding weight, for 2 days the mice had free access to both arms of the T-maze and were allowed to consume all reward in each arm of the maze before being returned to the start arm. Sweetened condensed milk (Carnation) (diluted 50:50 with water) was used as the reward, 0.1 ml in the feeding well of one arm [high reward arm (HR)], and 0.03 ml in the other [low reward arm (LR)]. For half of the mice, the HR arm was set to the right, and for the other half, the HR was set to the left.

Mice were habituated to the maze (with free-access to both arms) for three consecutive days, five trials run each day. They were cycled in their cage groups, leaving an inter-trial interval of approximately 5 min. For the next 3 days the trials were “forced”: access to one of the goal arms was blocked, thus forcing the mouse to sample a particular arm on each trial. The LR/HR order of the forced trials was determined pseudo-randomly so that the mice did not have more than two consecutive turns to either side. Mice ran 10 trials per day during this phase. During the final phase, the mice were allowed a choice of arms in each trial, but they were removed from the maze after eating the reward in the first selected arm. The first two trials for each mouse were forced and then followed by an additional 8 trials.

When the mice reached a criterion of selecting the HR arm on >90% of occasions during a session, a 5 cm barrier was introduced in the HR arm. For the first five trials with the barrier, the mice were only removed from the maze once they had climbed the barrier and eaten the large reward. Thereafter, and for all subsequent training, trials were performed with a schedule of 2 forced and 8 free choices and the mice were removed from the maze immediately after consuming the food in chosen arm. After three days with the 5 cm barrier, the barrier size in the HR arm was increased to 10 cm for a further three days. All subsequent testing used the 10 cm barrier.

After the habituation and training phase, the mice were divided in three groups. The Experimental group (Exp, n = 8) was tested on the effort-based T-maze decision-making task after following a run protocol (described below) under three levels of fatigue: low (40% of peak workload, PW), medium (60% PW) and high (80% PW). The Control Decision group (CtrlDec, n = 8) was tested in the same effort-based T-maze decision-making task as the Experimental group, but without following the run protocol (i.e., without being “fatigued”). Finally, the Control Run group (CtrlRun, n = 4) followed the same run protocol as the Exp group, but was not tested in the effort-based T-maze decision-making task (this group was used for analyses of neurotransmitters, not behaviour; see Experiment 4 below).

### Experiment 1

Experiment one was designed to assess the effects of fatigue on mice’s choice strategies. The behaviour of mice in the Exp group (n = 8) was tested on a T-maze effort-based decision-making task. All testing protocols were like the one devised by Walton *et al*.^18^ and included a T-maze with a choice between HR vs. LR, with a single barrier of 10 cm in the HD arm. The first two trials every day were forced in opposite directions, so that the mice was able to sample the reward in both arms before beginning the test trials. All mice then ran 8 test trials per day.

Every day, before the T-maze choice task, all mice followed a run protocol: they ran a (single) training session in a treadmill in order to induce a controlled level of fatigue. Each session started with a warm-up period of 10 min at 6 m/min, after mice ran for 40 min to a constant training load identified as a percentage of maximal speed achieved during an incremental load test. During the first 3 days, a single constant-load session was performed with intensity 40% of PW. After 1 day of recovery (without run or testing in the T-maze), the treadmill velocity was increased to 60% of PW for 3 days; and successively, again after 1 day of recovery, the treadmill velocity was increased to 80% of PW.

Thus, overall, the full experimental protocol followed by the Exp group every day (referred below as a “testing block”) combines a run protocol (with three different levels of fatigue - low (40%), medium (60%), and high (80%) - in successive days), followed immediately by the T-maze effort-based decision-making task. This procedure covered a period of up to 12 days.

The Control Decision group (CtrlDec, n = 8) was tested on a T-maze effort-based decision-making task, too, but without the run protocol. To match the duration of the experiment and exposure to the treadmill of the Exp group, mice in the CtrlDec group were placed in the treadmill for 40 min, too, but with a velocity of 0.5 m/s (less than normal walking velocity), which does not induce fatigue.

In keeping with the hypothesis that mice use a flexible form of cost-benefit analysis for their decision, we expected mice under high levels of fatigue (80% of PW) to change their choice from HR to LR, from the very first trial.

### Experiment 2

In order to assess whether the behavioural strategy used for effort-based decisions under high levels of fatigue (80% of PW) was a flexible form of cost-benefit analysis or a sort of bias (e.g., a learned aversion for the HR option), we conducted a second experiment: a re-test of the Exp group with treadmill velocity of 60% of PW, performed after Experiment 1 (with 1 resting day between Experiments 1 and 2). The rationale was that, in the presence of a flexible strategy, mice should revert to the same pattern of choice as they displayed *before* they experienced the high level of fatigue (80%); the opposite prediction could be made in the presence of a learned aversion for HR.

### Experiment 3

To determine whether fatigue was influencing the cost-benefit calculation or was simply preventing animals from being able to climb the barrier - either through physical exhaustion or by causing Pavlovian aversion to the barrier - we conducted a third experiment after Experiment 2 (with 1 resting day between Experiments 2 and 3). In this experiment, mice of the Exp group had to pay an effort cost to get either reward (“double-barrier” task). The protocol was identical to the previously described experiments, except that a 10 cm barrier was placed in *both* the HR and LR arms. Before the task, Exp group performed a training session in a treadmill with velocity of 80% of PW.

### Experiment 4

The fourth experiment was a re-test of the Exp group in high fatigue condition (80% of PW), performed after Experiment 3 (with 1 resting day between Experiments 3 and 4). This experiment was only performed for a single day.

### Analysis of brain tissue levels of dopamine and serotonin

Immediately after sacrifice, the brains of the animals in the Exp (n = 8), CtrlDec (n = 8) and CtrlRun (n = 4) groups was rapidly removed. The nucleus accumbens, hippocampus, midbrain and frontal cortex were subsequently dissected and homogenized in an ice bath for 2 min with Potter-Elvehjem homogenizer in 1 ml of 0.05N perchloric acid containing 0.004% sodium EDTA and 0.010% sodium bisulfite. The homogenate was 2-fold diluted in chromatographic mobile phase and centrifuged at 4,500 × *g* for 10 min. The supernatant was filtered on 0.45 μm PTFE sterile filters (Whatman) and directly injected for HPLC. Neurotransmitter recovery was satisfactory (≥90%) and reproducible, with percentage relative standard deviation ≤10%. The HPLC apparatus consisting of a Jasco (Tokyo, Japan) PU-2080 chromatographic pump and an ESA (Chelmsford, MA, USA) Coulochem III coulometric detector, equipped with microdialysis cell (ESA-5014b) porous graphite working electrode and solid state palladium reference electrode. The analytical conditions for biogenic amine identification and quantification were selected as previously reported^65^.

Brain areas were determined according to the mouse brain atlas^66^. According to the recognized ethical principles of “Replacement, Refinement and Reduction of Animals in Research”, in preliminary experiments performed in the same laboratories (University of Chieti) on euthanized control mice of the same age and weight, midbrain, nucleus accumbens, hyppocampus and prefrontal cortex place has been confirmed through post-mortem stereotaxic injection of dye (Evans blue 0.5% and Zelatin 5%) and histological examinations of the frozen sections.
